# Wheat Consumption Leads to Immune Activation and Symptom Worsening in Patients with Familial Mediterranean Fever: A Pilot Randomized Trial

**DOI:** 10.3390/nu12041127

**Published:** 2020-04-17

**Authors:** Antonio Carroccio, Pasquale Mansueto, Maurizio Soresi, Francesca Fayer, Diana Di Liberto, Erika Monguzzi, Marianna Lo Pizzo, Francesco La Blasca, Girolamo Geraci, Alice Pecoraro, Francesco Dieli, Detlef Schuppan

**Affiliations:** 1 Department of Health Promotion Sciences, Maternal and Infant Care, Internal Medicine and Medical Specialties (PROMISE), University of Palermo, 90124 Palermo, Italy; pasquale.mansueto@unipa.it (P.M.); maurizio.soresi@unipa.it (M.S.); francesca.fayer@gmail.com (F.F.); francescolablasca@gmail.com (F.L.B.); 2 Central Laboratory of Advanced Diagnosis and Biomedical Research (CLADIBIOR), University of Palermo, 90129 Palermo, Italy; diana.diliberto@unipa.it (D.D.L.); lopizzomarianna@gmail.com (M.L.P.); francesco.dieli@unipa.it (F.D.); 3 Institute of Translational Immunology and Research Center for Immunotherapy, University Medical Center, Johannes Gutenberg University, 55122 Mainz, Germany; erika.monguzzi@gmail.com; 4 Surgery Department, University of Palermo, 90129 Palermo, Italy; girolamo.geraci@unipa.it; 5 Hematology Unit for Rare Diseases, Laboratory of Molecular Genetic, Villa Sofia-Cervello, 90146 Palermo, Italy; a.pecoraro@villasofia.it; 6 Department of Biomedicine, Neurosciences and Advanced Diagnostics (BIND), University of Palermo, 90129 Palermo, Italy; 7 Division of Gastroenterology, Beth Israel Deaconess Medical Center, Harvard Medical School, Boston, MA 02215, USA

**Keywords:** AIDAI score, amylase trypsin inhibitor, non-celiac wheat sensitivity, CD14 lymphocytes, interleukin-1beta, tumor necrosis factor-α

## Abstract

We have identified a clinical association between self-reported non-celiac wheat sensitivity (NCWS) and Familial Mediterranean Fever (FMF). Objectives: A) To determine whether a 2-week double-blind placebo-controlled (DBPC) cross-over wheat vs. rice challenge exacerbates the clinical manifestations of FMF; B) to evaluate innate immune responses in NCWS/FMF patients challenged with wheat vs. rice. The study was conducted at the Department of Internal Medicine of the University Hospital of Palermo and the Hospital of Sciacca, Italy. Six female volunteers with FMF/NCWS (mean age 36 ± 6 years) were enrolled, 12 age-matched non-FMF, NCWS females, and 8 sex- and age-matched healthy subjects served as controls. We evaluated: 1. clinical symptoms by the FMF-specific AIDAI (Auto-Inflammatory Diseases Activity Index) score; 2. serum soluble CD14 (sCD14), C-reactive protein (CRP), and serum amyloid A (SSA); 3. circulating CD14^+^ monocytes expressing interleukin (IL)-1β and tumor necrosis factor (TNF)-α. The AIDAI score significantly increased in FMF patients during DBPC with wheat, but not with rice (19 ± 6.3 vs. 7 ± 1.6; *p* = 0.028). sCD14 values did not differ in FMF patients before and after the challenge, but were higher in FMF patients than in healthy controls (median values 11357 vs. 8710 pg/ml; *p* = 0.002). The percentage of circulating CD14^+^/IL-1β^+^ and of CD14^+^/TNF-α^+^ monocytes increased significantly after DBPC with wheat vs. baseline or rice challenge. Self-reported NCWS can hide an FMF diagnosis. Wheat ingestion exacerbated clinical and immunological features of FMF. Future studies performed on consecutive FMF patients recruited in centers for auto-inflammatory diseases will determine the real frequency and relevance of this association.

## 1. Introduction

Non-celiac wheat sensitivity (NCWS) is defined as a condition of self-reported symptoms after ingestion of wheat or other gluten-containing foods, after exclusion of celiac disease (CeD), and IgE-mediated wheat allergy [1]. NCWS is characterized by intestinal or extra-intestinal symptoms, such as fatigue, headache, or joint pain that improve on a wheat (gluten)-free diet [2]. The diagnosis is established when symptoms re-occur after exposure to wheat in a double-blind placebo-controlled (DBPC) challenge [3]. Its pathogenesis, although still debated, appears to be based on a prevalent activation of the innate immune system [4].

Familial Mediterranean fever (FMF) is an auto-inflammatory disease, due to an autosomal recessive mutation in the pyrin gene. This predisposes patients to unpredictable attacks of abdominal pain, fever, and malaise, caused by serositis, mainly peritonitis and pleuritis. FMF usually manifests as short and irregular attacks which spontaneously resolve within 2–3 days. Abdominal pain and fever are the most frequent symptom of FMF. Small bowel mucosal damage has been demonstrated by capsule endoscopy in 50% of FMF patients [5]. Therefore, FMF patients are frequently first seen by gastroenterologists in search for a diagnosis. Besides the above-mentioned genetic basis of FMF, emotional stress, viral disease, or menstruation can trigger bouts of the disease. FMF pathogenesis is linked to an overshooting generalized innate immune response that is dominated by interleukin-1β (IL-1β) and tumor necrosis factor-α (TNF-α), and characterized by a generalized serositis [6]. Current treatment of choice is regular oral colchicine that suppresses excessive monocyte activation [7]. 

In the past years, we observed an association between NCWS and FMF. Patients with self-reported symptoms due to wheat ingestion who came to our attention had a final diagnosis of FMF associated to NCWS (FMF/NCWS). 

In this pilot study, 6 patients with FMF/NCWS were subjected to a DBPC wheat vs. rice challenge, A) to assess clinical disease activity; and B) to study circulating and serum markers of systemic (innate) immune activation, compared to other patients with NCWS and healthy controls (HC). These limited data might represent a rationale for future definitive trials to assess the real prevalence of wheat-related symptoms and the putative role of wheat-free diet in FMF patients.

## 2. Materials and Methods

### 2.1. Patients

Between January 2015 and December 2017, a total of 22 patients received a new FMF diagnosis according to the Tel Hashomer Medical Center criteria [8] at the Department of Internal Medicine of the University Hospital of Palermo and of the Hospital of Sciacca, Italy. 

**Figure 1:**
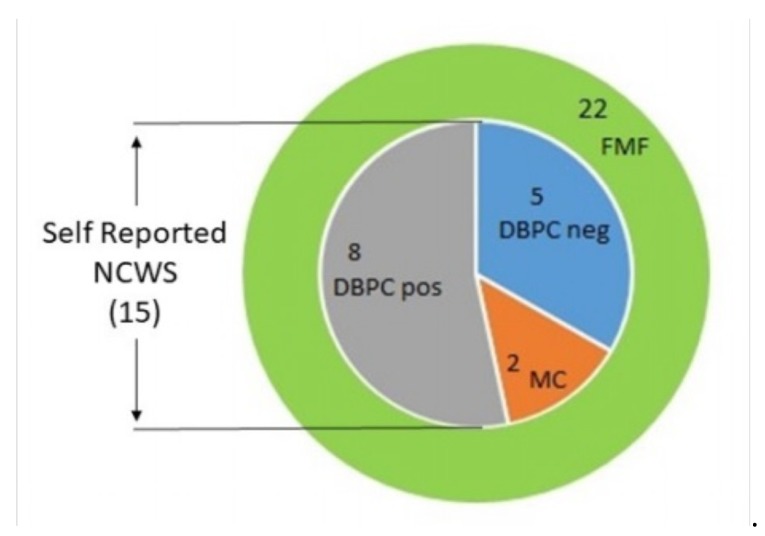
Number of self-reported non-celiac wheat sensitivity (NCWS) patients and of patients diagnosed with a “gluten-related” disease (celiac disease or NCWS), among 22 consecutive patients diagnosed with Familial Mediterranean Fever (FMF) in the 2 centers involved in the study. DBPC= double blind placebo-controlled challenge.

Fifteen of them self-reported symptoms due to wheat ingestion (68%), whereas 7 did not report symptom exacerbation related to wheat (Figure 1). 

Of the 15 patients reporting symptoms on wheat ingestion, 2 patients received a definitive diagnosis of CeD and 5 had a negative DBPC wheat challenge. The remaining 8 FMF patients (36% of the whole FMF group) were confirmed as FMF/NCWS patients by the DBPC wheat challenge. Of these, 6 accepted to enroll in this study (all females, mean age 36 ± 6 years), whereas 2 patients refused, as they did not accept a re-exposure to wheat ingestion. The 6 FMF patients included were on a wheat-free diet for 12–48 months (median 18) before enrollment and accepted to undergo a second DBPC wheat challenge between January and June 2019.

Exclusion criteria were: (a) age <18 years; (b) refusal to reintroduce wheat in the diet; (c) corticosteroids or non-steroidal anti-inflammatory drugs in the 2 weeks before biologic sample collection (blood and rectal mucosal biopsies); (d) presence of other “organic” gastrointestinal diseases; (e) pregnancy; (f) infectious diseases.

Two different control groups were recruited in the same centers. The first was composed of 12 patients (all females, mean age 35 ± 9 years), confirmed as NCWS by means of DBPC wheat challenge, who were not affected by FMF and/or other gastrointestinal diseases. They served to evaluate how far there may be differences in the inflammatory monocyte/cytokine patterns between NCWS subjects in a symptomatic phase (when they were consuming a wheat-containing diet) and the FMF/NCWS patients on the wheat challenge. They were chosen at random from patients who were enrolled in other studies on NCWS. All were matched to the FMF patients for sex and age. These subjects were symptomatic (mainly abdominal symptoms, resembling irritable bowel syndrome) on the DBPC wheat challenge. Eight healthy subjects HC, who underwent colonoscopy for colon carcinoma screening, as first-degree relatives were affected, served as additional sex- and age-matched controls. Clinical characteristics of FMF/NCWS patients are given in Supplemental file 1 (Table S1).

### 2.2. Methods

NCWS was diagnosed as previously described [9], with patients meeting the proposed criteria [1]. Other gastrointestinal diseases, in particular CeD, inflammatory bowel disease (IBD), and IgE-mediated wheat allergy, were carefully excluded as follows. 

Exclusion of CeD:

Before evaluation for NCWS, patients were instructed to eat foods containing wheat, consuming the equivalent of at least 5 slices of wheat bread per day (about 12 grams of gluten) for 4 weeks. At the end of this period, all patients underwent serum testing for antibodies to tissue transglutaminase (anti-tTG) IgA, antibodies to deamidated gliadin peptides (anti-DGP) IgG, and antibodies to gliadin (AGA) IgA and IgG, as measured using commercial kits (Eu-tTG IgA, and anti-gliadin IgA and IgG, Eurospital Pharma, Trieste, Italy; Quanta-Lite Gliadin IgG II, Inova Diagnostics, San Diego, CA, USA). Patients were also typed for HLA-DQ2/8 phenotypes by polymerase chain reaction, using sequence-specific primers with a rapid detection method (DQ-CD Typing Plus kit by BioDiaGene, Palermo, Italy). Patients positive for the DQ2 and/or the DQ8 haplotypes also underwent duodenal mucosal biopsy, regardless of the results of the CeD-specific antibody assay. 

CeD diagnosis was excluded when: A) DQ2 and/or DQ8 haplotypes were absent, or B) anti-tTG IgA and anti-DPG IgG were negative and duodenal histology showed a normal villus/crypt ratio (≥3).

Furthermore, CeD diagnosis was considered likely if patients were positive for anti-endomysial antibodies (EmA) in the medium of cultured duodenal biopsies, even if the villus/crypt ratio in the duodenal mucosa was normal. Consequently, these patients were not included in the NCWS group.

Exclusion of IBD:

IBD diagnosis was excluded when serum C-reactive protein (CRP), erythrocyte sedimentation rate, and white blood cell count were normal in repeated examinations, performed when the patients were symptomatic. Furthermore, all patients underwent abdominal ultrasound evaluation of the intestine, and those with ultrasound signs of suspected IBD were excluded. Patients with a clinical history of suspected IBD (i.e., presence of rectal bleeding or hematochezia) also underwent a complete ileo-colonoscopy. IBD diagnosis was excluded in those whose endoscopy and histology were negative.

Exclusion of IgE-mediated wheat allergy:

IgE-mediated wheat allergy was excluded by negative IgE-serum titers for wheat and/or negative skin prick test for wheat.

Elimination diet and DBPC food challenge for NCWS diagnosis:

After exclusion of CeD, IBD, IgE-mediated wheat allergy, and other gastrointestinal diseases, the patients underwent the DBPC wheat challenge for NCWS diagnosis, according to our established protocol [9]. In brief, before final NCWS diagnosis, all patients were on a standard elimination diet, which excluded wheat, cow’s milk, eggs, tomato, and chocolate. Patients with self-reported food hypersensitivity excluded ingestion also of other food(s) causing symptoms. Food diaries were kept during the elimination diet, to assess dietary intake and adherence to the diet. 

After 4 weeks of the elimination diet, DBPC challenges were performed according to a computer-generated sequence determined by an observer not involved in the study. For confirmation of NCWS, patients received sachets, coded A or B, containing 80 g of wheat or rice flour, respectively. Sachets A or B were given once daily, at dinner, for 2 consecutive weeks, and then after 1 week of washout, patients received the other sachets for another 2 weeks (cross-over design). If needed, the washout period was, eventually, extended for a maximum of another 2 weeks, until the symptoms induced by the previous challenge had completely resolved, before starting the next challenge. 

Wheat or rice flour, given in sachets, was consumed after cooking, as determined by the patients themselves, and there was no overt difference in their appearance. Physicians assessing outcomes (AC, PM, FLB, MS) were blinded in respect to the flours ingested (wheat or rice). Wheat sachets contained 6.5 g of gluten and an estimated 0.3 g of amylase trypsin inhibitors (ATIs), as determined by bioassay [10]. The codes of the sachets were broken only at the end of the study, and the investigators did not know their contents during the study period. Challenges for other foods in patients with suspected multiple food hypersensitivities were performed in an open fashion.

During all phases of the evaluation, the severity of symptoms was recorded: patients completed a 10 point visual analog scale (VAS, with 0 representing no and 10 intolerable symptoms), which assessed the following overall symptoms and the following specific symptoms: abdominal pain or discomfort, abdominal distension, bloating, increased flatus, diarrhea (increased passage and/or urgent need for defecation of loose stools), constipation (decreased passage of stools or feeling of incomplete evacuation), heartburn, acid regurgitation, nausea and vomiting. Extra-intestinal symptoms were also recorded: rash/dermatitis, headache, foggy mind, fatigue, fainting, numbness of the limbs, joint/muscle pains, oral/tongue lesions, or other specific symptoms reported by the individual patient. 

The challenges were stopped when severe clinical reactions occurred for at least 2 consecutive days (increase in VAS score >30% over the basal value) for intestinal and/or extra-intestinal symptoms. Challenges were considered positive and NCWS confirmed when the same symptoms that had been initially present initially, reappeared, after their disappearance on the elimination diet, on the wheat flour challenge and when the VAS score was >30% over the basal values. 

DBPC wheat challenge performed in the present study:

The challenge protocol used for the present study was almost identical to that described above for establishing the diagnosis of NCWS. At study entry, all patients were on a wheat-free diet. Randomization and DBPC wheat vs. rice flour challenge were performed as described above. The symptoms for the Auto-Inflammatory Disease Activity Index (AIDAI) score were recorded by the patients themselves on the scoring sheet; clinical reactions were also evaluated by 4 of the authors (AC, PM, FLB, MS). 

FMF clinical evaluation:

A modified version of the AIDAI was used to assess disease activity in FMF [11]. AIDAI is a validated instrument designed to standardize the assessment of FMF clinical activity and to objectivate the FMF patients’ symptoms’ change across trials. In its current form, the AIDAI score is very easy to use by the patients themselves.

The ADAI score was calculated daily during the 2 weeks before the beginning of the challenge and during the periods of the DBPC challenge (2 weeks on wheat challenge and 2 weeks on placebo). AIDAI consists of 12 items: fever, overall symptoms, abdominal pain, nausea/vomiting, diarrhea, headaches, chest pain, painful nodes, arthralgia or myalgia, swelling of the joints, eye manifestations, skin rash. It was scored as yes (1 point) or no (0 point); therefore, the sum score on a single day can range between 0 to 12, and in the 14-day period, from 0 to 168 (see Supplemental file 2).

Blood sampling:

In FMF patients, venous blood samples were obtained immediately before the challenge. Additional blood samples were collected at the end of the two challenge periods. 

Blood samples of NCWS patients were obtained when they were symptomatic, on a wheat-containing diet, and from HC. 

Markers of inflammation:

The following serum parameters were determined by commercial ELISAs: soluble CD14 (sCD14) (R&D Systems), CRP (Roche Diagnostics S.p.A, Italia), and serum amyloid A (SAA). 

Isolation of peripheral blood mononuclear cells and flow cytometry analysis:

Peripheral blood mononuclear cell (PBMC) were isolated from heparinized blood by Ficoll-Hypaque (Sigma) density-gradient centrifugation (2000 rpm for 20 min). For gating strategy see Supplemental file 3. 

### 2.3. Statistical Analysis

Sample size was not calculated since it was a pilot study including a few number of patients. Gaussian values were expressed as mean ± SD. For parameters with non-Gaussian distribution, values were expressed as range and median. Differences between the groups were calculated using the Kruskall–Wallis test, applying the Mann–Whitney U test for significant variables. To compare the values of the AIDAI score, the Friedman test was used and, if significant, the Wilcoxon rank sum test. To evaluate the correlation between the serum parameters of inflammation, the Pearson’s correlation coefficient was applied.

All data were analyzed using SPSS version 22.0 (SPSS Inc, Chicago, IL, USA) and MedCalc Software (Mariakerke, Belgium).

The study was approved by the Ethics Committee of the University of Palermo, Italy. Informed consent was obtained from all patients who participated in the study.

## 3. Results

### 3.1. Clinical Data

During the study period, a total of 995 outpatients with self-reported symptoms on wheat ingestion were examined at the 2 centers. FMF was newly diagnosed in 15 (14 females), equivalent to a prevalence of 1.5% in this population. FMF diagnosis had been previously missed in all these patients.

After excluding 2 patients who were diagnosed with CeD, and 5 patients who tested negative in the DBPC wheat challenge, 8 FMF/NCWS patients were identified. Six of them accepted to enroll in the present study. 

These 6 patients tested negative for serum anti-tTG IgA and anti-DGP IgG; four of them tested negative for the HLA DQ2 and DQ8 haplotypes. The 2 HLA DQ2 and DQ8 positive patients underwent duodenal biopsies to exclude a seronegative CeD, and both had normal villi/crypts ratio (>3:1).

All patients were Caucasian, belonged to the Mediterranean population of Southern of Italy, and all carried one of the FMF gene sequence variants, located on chromosome 16.p13.3, encoding the protein pyrin, recorded in Infevers, an online registry of FMF genetic variants. They complained mainly of abdominal pain and diarrhea, and of increased symptoms on wheat ingestion. None of them had been diagnosed with FMF before their referral to us. All had undergone at least one recent gastroscopy and colonoscopy, and CeD, IBD, and IgE-mediated wheat allergy were excluded. 

At study start, 4 patients were on colchicine treatment and reported improvement but no resolution of their FMF symptoms; two patients were only on a diet that excluded wheat, as they were asymptomatic on this regimen.

The modified AIDAI score was recorded for 2 weeks before the DBPC challenges, ranging between 4 and 8 points (median 5.5), as none of the patients were completely asymptomatic.

**Figure 2:**
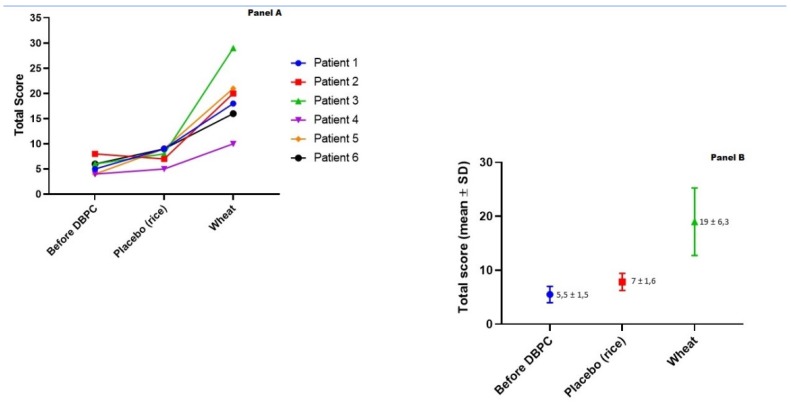
Individual Auto-Inflammatory Diseases Activity Index (AIDAI) scores at baseline and after the placebo and wheat challenges in 6 patients with FMF and NCWS (**A**), and mean values ( ± SD) at baseline and after the placebo and the wheat challenge (**B**).

Three patients were initially randomly assigned to wheat and 3 to rice (placebo), to shifted to the other regimen according to the cross-over design. Figure 2A shows the individual AIDAI scores at baseline (on a wheat-free diet) and after the wheat vs. placebo challenges. Under the placebo challenge, the AIDAI score increased slightly (from 5.5 ± 1.5 to 7.0 ± 1.6, mean ± SD). Under the wheat challenge, however, the score showed a marked increase to 19 ± 6.3 (Figure 2B). 

The difference between the placebo (rice) and the wheat challenge, and between baseline and the wheat challenge were significant (both *p* = 0.028). On the wheat challenge, 3 patients reported fever and 2 did not complete the 2 weeks of the challenge (pts 4 and 5, stopping after 2 and 5 days, respectively), as they developed severe symptoms (fever, diarrhea, vomiting, headache, arthro-myalgias, and skin rash) starting on the first day of wheat consumption. The AIDAI score of these patients was the highest of the whole group, and their scores were included as the last observation put forward (intention-to-treat statistical analysis). The other 4 patients completed the two 14-day challenges, despite increased symptoms during the challenge that finally turned out to be with wheat. In general, symptoms occurred within 1 and 8 days after beginning the wheat challenges (median 3 days). The individual AIDAI score and each sub-score are shown in Supplemental file 3 (Figure S1).

### 3.2. Serum Markers of Inflammation

Table 1 shows the median and range of sCD14, CRP, and SAA. Mean CRP and SAA serum levels were increased (almost) twofold in FMF patients after the wheat challenge, but this did not reach statistical significance. Compared to HC, FMF patients (before and after the wheat challenge), as well as non-FMF NCWS patients on a wheat-containing diet, showed significantly higher values of sCD14. Considering the whole study population, CRP correlated with SAA (*r* = 0.856; *p* < 0.0001) and with sCD14 (*r* = 0.415; *p* = 0.01).

### 3.3. Immune Profiling of PBMC by FACS 

**Figure 3:**
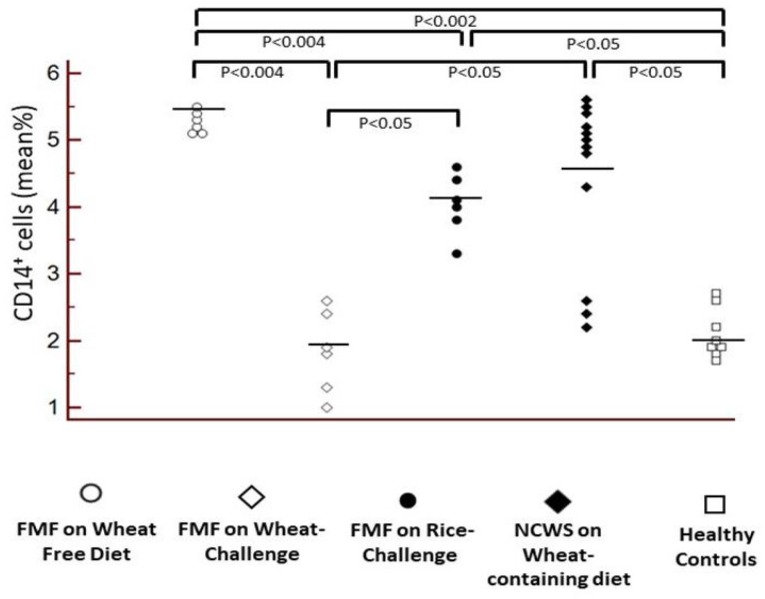
Evaluation of the percentage of CD14^+^ monocytes in the peripheral blood of the 6 FMF patients with NCWS, before and after the wheat challenge, and after the placebo (rice) challenge, in twelve symptomatic NCWS patients (on a wheat containing diet), and in 8 healthy controls. Symbols indicate the individual values; bars indicate mean values.

The percentage of total CD14^+^ PBMC was similar in FMF patients before the wheat challenge, on wheat-free diet, and in symptomatic NCWS patients on a wheat-containing diet, and significantly higher in both groups compared to the HC (for FMF *p* = 0.002, for NCWS *p* = 0.05). Surprisingly, and in line with the results for serum sCD14 (Table 1), peripheral CD14^+^ cell counts declined in FMF patients after vs. before wheat challenge (*p* = 0.004); the values after the wheat challenge were also significantly lower than after the placebo challenge (*p* = 0.05) (Figure 3). 

**Figure 4:**
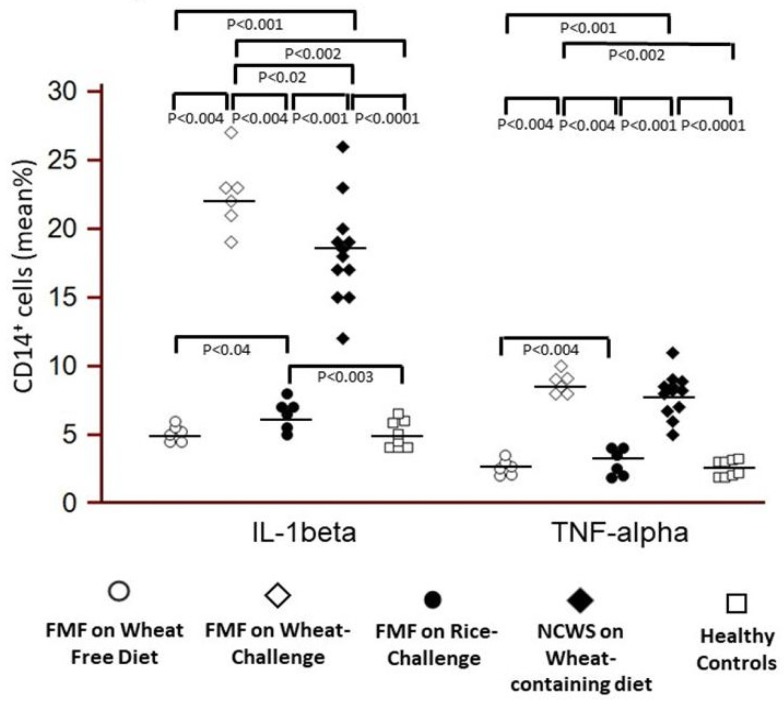
Evaluation of CD14+/IL1-beta+, and CD14+/TNF+ monocytes in the peripheral blood of the 6 FMF patients with NCWS, before and after the wheat challenge and placebo (rice) challenge, in 12 symptomatic NCWS patients (on a wheat containing diet) and in 8 healthy controls.

However, compared to baseline, the percentage of circulating pro-inflammatory CD14^+^/IL-1β^+^ monocytes was significantly increased in FMF patients after the wheat challenge (*p* = 0.004), with values significantly higher than after the placebo challenge (*p* = 0.004) and HC *p* = 0.02). A comparable pattern was seen for CD14^+^/TNF-α^+^ monocytes (*p* = 0.004 vs. baseline, *p* = 0.004 vs. placebo challenge, *p* = 0.002 vs. HC) (Figure 4).

Interestingly, the 2 patients who did not complete the 2 weeks of the wheat challenge, as they developed severe symptoms and showed the highest AIDAI score of the whole group, were those with the highest percentage of circulating pro-inflammatory CD14^+^/IL-1β^+^ monocytes (Figure 4).

## 4. Discussion

Although non-celiac gluten sensitivity (NCGS, correctly NCWS) had been described as a distinct clinical condition about 40 years ago [12], it is now identified as a syndrome, characterized by symptoms which can involve the gastrointestinal tract, the nervous system, the skin, the female reproductive tract, and other organs, following the ingestion of gluten/wheat, in subjects who do not suffer from CeD or IgE-mediated wheat allergy [13,14,15]. 

However, NCWS is a still ill-defined clinical condition of wheat sensitivity in patients in whom CeD or IgE-mediated wheat allergy have been excluded. Other diseases can overlap with NCWS, in particular some autoimmune and auto-inflammatory conditions that are worsened by wheat ingestion and improve on a wheat (“gluten”)-free diet. In this respect, it has been demonstrated that one third of NCWS subjects showed associated autoimmune diseases (such as Hashimoto’s thyroiditis) and more than 40% had positive serum anti-nuclear antibodies compared to 2%–6% in the control group including subjects suffering from irritable bowel syndrome [16]. 

The frequency of FMF among our patients with self-reported NCWS was 1.5%, which is astonishingly high compared to the worldwide prevalence of FMF, estimated at 1:100,000–150,000, with the highest prevalence in non-Ashkenazi Jews or Armenians, estimated at 1:250–500, groups, that were not represented in our cohort [17]. Therefore, in populations with a higher, but also with a lower prevalence of FMF, we need to consider the diagnosis of NCWS combined with FMF, an otherwise rare disease that is often overlooked in clinical practice. 

It must be underlined that FMF diagnosis had been missed previously in all the patients included in our study; this could be due to the rarity of this disease and the consequent low awareness by physicians. Therefore, both internists and gastroenterologists should consider this diagnosis also in patients with self-reported NCWS and abdominal and general inflammatory symptoms.

During the DBPC challenge of the present study, all the enrolled patients relapsed on the wheat challenge, to show an AIDAI score significantly higher with the wheat challenge than at baseline or with the placebo challenge. Symptom relapse was so severe that 2 patients interrupted the challenge on the 3rd and 6th day, respectively, and 3 patients developed fever (>38°C). None of them reacted to the placebo (rice) challenge.

We found that serum levels of sCD14 were significantly higher in FMF-NCWS patients than in HC, with levels comparable to patients with NCWS alone. In FMF-NCWS patients, sCD14 levels did not increase after the wheat challenge compared to baseline, suggesting a stable pro-inflammatory predisposition and, perhaps, a tissue recruitment towards serosal tissues after wheat exposure. Notably, the number of circulating CD14^+^/IL-1β^+^ and CD14^+^/TNF-α^+^ monocytes increased significantly in the FMF patients 12 h after the wheat challenge, compared with baseline and with the placebo (rice) challenge. In view of decreased total CD14+ cells, this suggests a dramatic increase in the proportion of pro-inflammatory vs. non-activated monocytes in the circulation, considered a hallmark of (auto-) inflammation. Their values after the wheat challenge were also significantly higher than in HC. Notably, increased IL-1β and TNF-α^+^ production by monocytes are considered hallmarks of innate immune activation and consequent inflammation in FMF [18], and possibly also in the pathogenesis of NCWS [19].

Our findings are in accord with prior reports that stressed the impact of environmental factors on the course of FMF [20], although no defined triggers had been identified so far. 

The findings of the present study are well in agreement also with our prior experimental data that showed that a specific non-gluten protein component of wheat, the family of ATIs, activate innate immunity in the gut [21,22]. ATIs engage the toll-like receptor 4 (TLR4)-MD2-CD14 complex, leading to an upregulation of monocyte, macrophage, and especially dendritic cell maturation markers and an increased release of pro-inflammatory cytokines and chemokines by these myeloid cells [23]. Furthermore, it has been demonstrated that a wheat-, and therefore, ATI-containing diet worsened IBD, as well as nutritional and inhalative allergies [10,21,24]. Moreover, our unpublished results in mouse models of autoimmune diseases, such as multiple sclerosis or systemic lupus erythematosus, indicate that nutritional ATIs worsen ongoing chronic inflammatory diseases in general [25]. In the mentioned studies, IL-1β and TNF-α^+^ were central mediators of myeloid cell activation that was triggered in the intestine (and the periphery) by wheat ATIs. We therefore suggest that ATIs may be the primary “culprits” in wheat that activate innate immunity and exacerbates FMF [21,22,23].

Our study has some limitations. First, it is a pilot study, including a low number of patients. However, FMF is a rare disorder and we are planning further studies, involving a much higher number of FMF patients, and considering a broad range of biological parameters. The completion of the planned studies, however, will take several years and we consider it unethical to withhold publication of the present study that shows a spectrum of significant results, despite the low number of patients.

Second, we observed the association between wheat consumption and FMF in 2 tertiary centers that are dedicated to wheat and nutrition-related diseases. Consequently, the high frequency of FMF patients with self-reported NCWS, and the association of FMF with NCWS may have been overestimated. Thus, we were not able to evaluate the overall therapeutic contribution of a wheat-free diet in FMF. 

Third, other wheat-related components and mechanisms may add to the worsening of intestinal symptoms in our FMF patients exposed to wheat. Such a mechanistically different inflammatory condition that causes abdominal symptoms could be “atypical” food allergies, prominently to wheat, suggested by recent histological [9] and confocal endomicroscopy studies [26,27]. 

Fourth, the intestinal microbiota, as affected by wheat compared to a wheat-free diet, may be an important environmental factor affecting the severity of FMF [28]. But here again, we could demonstrate the wheat ATI can directly promote intestinal pro-inflammatory dysbiosis [29]. 

Finally, we focused only on the role of IL-1β and TNF-α production by PBMC, but likely, there is also a role for IL-6, IL-17, IL-22, and other cytokines, as it has been suggested in other studies on FMF patients [30]. 

## 5. Conclusions

Our pilot study provides the first evidence that self-reported symptoms due to wheat ingestion can reveal a FMF diagnosis previously missed, and that wheat ingestion can lead to immune activation and exacerbation of FMF. The subgroup of FMF patients who can benefit from a wheat-free diet needs to be well-defined in future studies. In this subgroup of patients, it is possible that a wheat-free diet could become a cornerstone for treatment and prevention of FMF. In general, however, regardless of the patients’ subjective feelings about the severity of their fever and pain attacks, it is advisable to continue colchicine therapy at adequate doses to prevent the one life-threatening complications of FMF, such as amyloidosis.

## Figures and Tables

**Table 1:** Median and range of soluble CD14 (sCD14), C-reactive protein (CRP), and serum amyloid A (SAA) in the 6 FMF patients at baseline (on a wheat-free diet), at the end of the wheat challenge, and at the end of the placebo (rice) challenge, in 12 patients with symptomatic NCWS and in 8 healthy controls (both on a wheat-containing diet).

	sCD14 (pg/ml)	CRP (mg/L)	SAA (mg/L)
FMF at baseline	11357 (9215–14210)	2.6 (2–9)	6.9 (0.7–26.7)
FMF after wheat challenge	10023 (9112–1436)	5.0 (2–9)	17.3 (2.9–42.7)
FMF after placebo (rice) challenge	11035 (9068–13210)	3.6 (2–5)	12.1 (1.8–27,6)
NCWS patients (on wheat)	11089 (9043–12245)	2.9 (2–7)	6.7 (1.9–38.4)
Healthy controls	8710 (8023–9205)	2.6 (1–4)	4.7 (1.9–34.1)

For sCD14, Kruskall–Wallis test: *p* = 0.001; for other comparisons: Mann–Whitney U test: FMF at baseline vs. HC, *p* = 0.002; FMF after wheat challenge vs. HC, *p* = 0.002; FMF after placebo (rice) challenge vs. HC, *p* = 0.002; NCWS vs. HC, *p* = 0.0001. No other comparisons reached statistical significance.

## Supplementary Materials

The following are available online at https://www.mdpi.com/2072-6643/12/4/1127/s1, Table S1: Demographic and clinical characteristics of the 6 FMF patients included; file 2: AIDAI score form for the patients; file 3: Representative FACS plots showing the gating strategy used to analyze expression of the pro-inflammatory cytokines IL-1b and TNF-a by CD14+ monocytes obtained from the peripheral of a FMF patient on the wheat-free diet. Blue: isotype control mAb. Pink: cytokine-specific mAb; Figure S1: Individual ADAI scores of the FMF-NCWS patients at baseline, and after wheat or rice (placebo) challenge.

## Registration

The study was registered at Clinicaltrials.gov (registration number NCT03563300), accessible at: https://www.clinicaltrials.gov/ct2/show/NCT03563300?term=wheat&cond=Familial+Mediterranean+Fever&draw=2&rank=1).

## Abbreviations

AIDAI	Auto-Inflammatory Disease Activity Index
CeD	Celiac Disease
CRP	C-Reactive Protein
DBPC	Double-Blind Placebo-Controlled
FABP2	Fatty Acid-Binding Protein 2
FMF	Familial Mediterranean Fever
LBP	Lipopolysaccharide-Binding Protein
NCWS	Non-Celiac Wheat Sensitivity
PBMC	Peripheral Blood Monocytes Cells
SAA	Serum Amyloid A
sCD14	soluble CD14
SD	Standard Deviation
