# Supplementary material for: Wheat Consumption Leads to Immune Activation and Symptom Worsening in Patients with Familial Mediterranean Fever: A Pilot Randomized Trial
Source: Nutrients. 2020 Apr 17;12(4):1127. doi: 10.3390/nu12041127 (PMC7230718; doi:10.3390/nu12041127)
Supplement: Supplementary file 1 [file nutrients-12-01127-s001.zip › nutrients-731041-supplementary/Supplemental figure S1 Nutrients.docx]

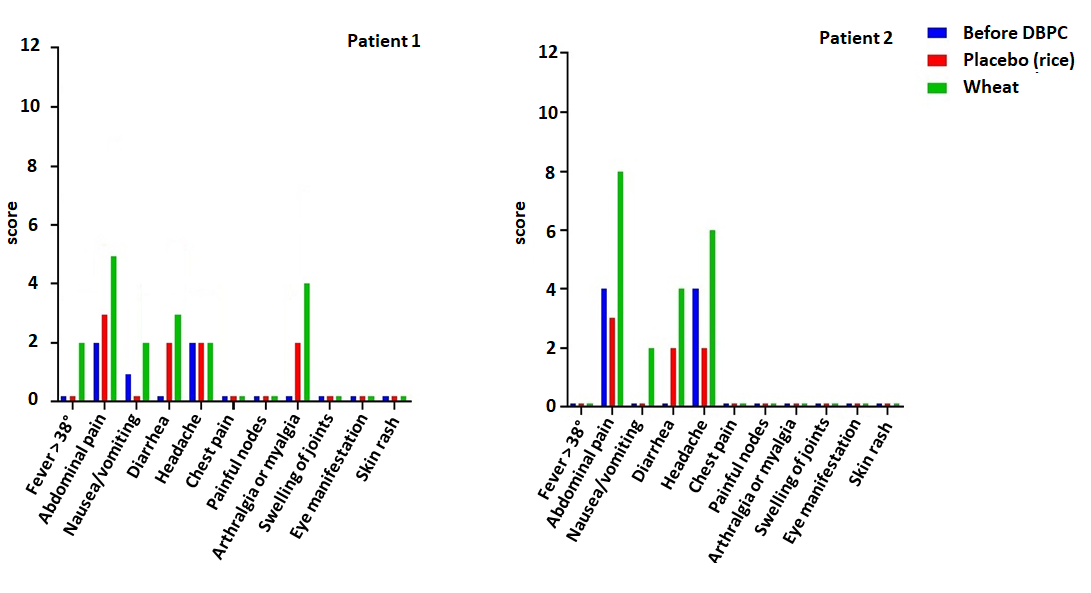


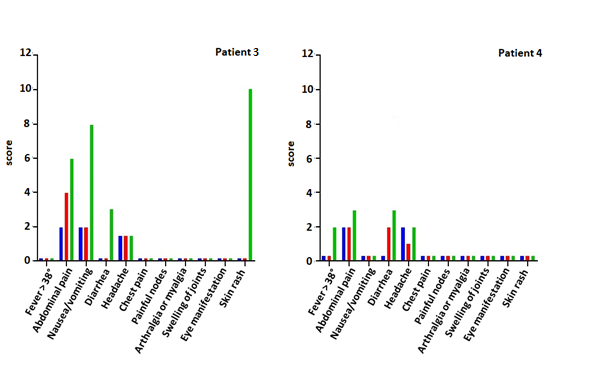


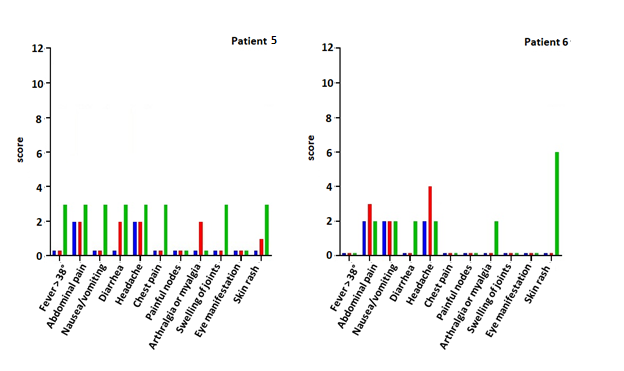


**Supplementary Fig.S1:** Individual ADAI scores of the FMF-NCWS patients at baseline, and after wheat or rice (placebo) challenge
