# Supplementary material for: Wheat Consumption Leads to Immune Activation and Symptom Worsening in Patients with Familial Mediterranean Fever: A Pilot Randomized Trial
Source: Nutrients. 2020 Apr 17;12(4):1127. doi: 10.3390/nu12041127 (PMC7230718; doi:10.3390/nu12041127)
Supplement: Supplementary file 1 [file nutrients-12-01127-s001.zip › nutrients-731041-supplementary/Supplemental file 2 Nutrients.docx]

| Name: …………………………………………………. | | | | | Age: | | | | Period: | | | | |
| --- | --- | --- | --- | --- | --- | --- | --- | --- | --- | --- | --- | --- | --- |
|  | Fever  >38°C | Overall  Symptoms | Abdominal  pain | Nausea/  Vomiting | Diarrhea | Head  aches | Chest  pain | Painful  nodes | | Arthralgia  or myalgia | Swelling  of  joints | Eye  manifestations | Skin  rash |
| Score | 0 or 1 | 0 or 1 | 0 or 1 | 0 or 1 | 0 or 1 | 0 or 1 | 0 or 1 | 0 or 1 | | 0 or 1 | 0 or 1 | 0 or 1 | 0 or 1 |
| Day |  | | | | | | | | | | | | |
| 1 |  |  |  |  |  |  |  |  | |  |  |  |  |
| 2 |  |  |  |  |  |  |  |  | |  |  |  |  |
| 3 |  |  |  |  |  |  |  |  | |  |  |  |  |
| 4 |  |  |  |  |  |  |  |  | |  |  |  |  |
| 5 |  |  |  |  |  |  |  |  | |  |  |  |  |
| 6 |  |  |  |  |  |  |  |  | |  |  |  |  |
| 7 |  |  |  |  |  |  |  |  | |  |  |  |  |
| 8 |  |  |  |  |  |  |  |  | |  |  |  |  |
| 9 |  |  |  |  |  |  |  |  | |  |  |  |  |
| 10 |  |  |  |  |  |  |  |  | |  |  |  |  |
| 11 |  |  |  |  |  |  |  |  | |  |  |  |  |
| 12 |  |  |  |  |  |  |  |  | |  |  |  |  |
| 13 |  |  |  |  |  |  |  |  | |  |  |  |  |
| 14 |  |  |  |  |  |  |  |  | |  |  |  |  |
| TOTAL |  |  |  |  |  |  |  |  | |  |  |  |  |
| TOTAL SCORE FOR THE TWO WEEKS PERIOD: | | | | | | | | | | | | | |

Auto-Inflammatory Disease Activity Andex (AIDAI). Patients were asked to record the absence (score = 0) or the presence (score = 1) of each of these symptoms related to FMF during 3 periods: (A) the two weeks before start of the DBPC challenge, (B) and (C) during the wheat and rice (placebo) DBPC challenge periods, respectively.
Each line refers to a day in the two weeks of each period A) to C).

The total score (sum of point for each item) in a single day ranges between 0 to 12. In the 14-day period, the cumulative score ranges from 0 to 168. Modified from Piram M et al. (ref. 12).
