# Supplementary material for: Wheat Consumption Leads to Immune Activation and Symptom Worsening in Patients with Familial Mediterranean Fever: A Pilot Randomized Trial
Source: Nutrients. 2020 Apr 17;12(4):1127. doi: 10.3390/nu12041127 (PMC7230718; doi:10.3390/nu12041127)
Supplement: Supplementary file 1 [file nutrients-12-01127-s001.zip › nutrients-731041-supplementary/Supplemental table S1.docx]

Demographic and clinical characteristics of the six FMF patients included.

|  | Pt 1 | Pt 2 | Pt 3 | Pt 4 | Pt 5 | Pt 6 |
| --- | --- | --- | --- | --- | --- | --- |
| Age (years) | 35 | 30 | 25 | 31 | 57 | 37 |
| FMF mutation | R202Q | Nt 1588-69 G>A | R202Q | R202Q | Nt 1588-69 G>A | Nt 1588-69 G>A  R202Q |
| DQ2 or DQ8 haplotypes | DQ2 | None | none | DQ8 | None | None |
| FMF major clinical criteria | Fever | Fever | Fever | Fever | None | Peritonitis |
| Colchicine treatment (dose and benefit) | 1 mg/day  Benefit | No treatment | 1 mg/day  Benefit | 2 mg/day  Benefit | No treatment | 1 mg/day  Benefit |
| Self-reported symptoms/signs  caused by wheat ingestion | Fever, sideropenic anaemia, abdominal pain, diarrhoea | Diarrhoea, abdominal distension and pain, vomiting, urticaria, weight increase | Fever, diarrhoea, abdominal distension and pain, cystitis | Fever, abdominal distension and pain, nausea, diarrhoea, headache,  fatigue, mental confusion, joint pain, eczema | Heartburn, urticaria, abdominal distension and pain, anal fissures, alternating bowel movements, weight loss | Abdominal distension and pain, constipation,  heartburn |
| Associated diseases | Autoimmune hepatitis | PCOS, arterial hypertension | None | Obesity, allergic asthma and rhinitis | Psoriasis, nickel allergy, fibromyalgia, GERD, gastric ulcer | Kidney lithiasis, recurrent salpingitis |
| Intolerance towards other foods | Wheat only | Wheat, cow’s milk and derivatives | Wheat, cow’s milk and derivatives | Wheat only | Wheat, cow’s milk and derivatives | Wheat only |

Abbreviations: PCOS=  Polycystic ovary syndrome; GERD= Gastro-esophageal reflux disease
